# The Novel Ubiquitin Ligase Complex, SCF^Fbxw4^, Interacts with the COP9 Signalosome in an F-Box Dependent Manner, Is Mutated, Lost and Under-Expressed in Human Cancers

**DOI:** 10.1371/journal.pone.0063610

**Published:** 2013-05-02

**Authors:** William W. Lockwood, Sahiba K. Chandel, Greg L. Stewart, Hediye Erdjument-Bromage, Levi J. Beverly

**Affiliations:** 1 Department of Medicine, Division of Hematology and Oncology, James Graham Brown Cancer Center, University of Louisville, Louisville, Kentucky, United States of America; 2 Cancer Biology and Genetics Section, Cancer Genetics Branch, National Human Genome Research Institute, National Institutes of Health, Bethesda, Maryland, United States of America; 3 British Columbia Cancer Research Center, Vancouver, British Columbia, Canada; 4 Molecular Biology Program, Memorial Sloan-Kettering Cancer Center, New York, New York, United States of America; University of Minnesota, United States of America

## Abstract

Identification of novel proteins that can potentially contribute to carcinogenesis is a requisite venture. Herein, we report the first biochemical characterization of the novel F-box and WD40 containing protein, FBXW4. We have identified interacting protein partners and demonstrated that FBXW4 is part of a ubiquitin ligase complex. Furthermore, the Fbxw4 locus is a common site of proviral insertion in a variety of retroviral insertional mutagenesis murine cancer models and Fbxw4 mRNA is highly expressed in the involuting murine mammary gland. To begin to characterize the biochemical function of Fbxw4, we used proteomic analysis to demonstrate that Fbxw4 interacts with Skp1 (SKP1), Cullin1 (CUL1), Ring-box1 (RBX1) and all components of the COP9 signalosome. All of these interactions are dependent on an intact F-box domain of Fbxw4. Furthermore, Fbxw4 is capable of interacting with ubiquitinated proteins within cells in an F-box dependent manner. Finally, we demonstrate that FBXW4 is mutated, lost and under-expressed in a variety of human cancer cell lines and clinical patient samples. Importantly, expression of FBXW4 correlates with survival of patients with non-small cell lung cancer. Taken together, we suggest that FBXW4 may be a novel tumor suppressor that regulates important cellular processes.

## Introduction

Ubiquitin ligase complexes catalyze the conjugation of ubiquitin onto substrate proteins [1]. Ubiquitination of proteins can have a variety of effects on protein function with the best well studied being the regulation of protein stability [2]. Ubiquitin ligase complexes (also called E3 ubiquitin ligases) interact with an E1 (ubiquitin activating enzyme), and an E2 (ubiquitin conjugating enzyme) [1]. The E1 and E2 enzymes are generally shared between many E3 ligases and the E3 component is the specificity factor that interacts directly with substrate proteins. One type of E3 ubiquitin ligase complex, the SCF ubiquitin ligase complex, contains Skp1, Cullin1, ring-box1 and any one of more than seventy F-box containing proteins encoded in the genomes of higher eukaryotes [3], [4]. The F-box domain is responsible for directly interacting with Skp1, whereas the other domains contained within the protein are responsible for interacting with and bringing substrate proteins into proximity of the ubiquitin ligase [5]. Importantly, multiple F-box containing proteins are well characterized to play direct roles in the genesis of human cancers [6], [7], [8], [9], [10]. For example, mutations that lead to a loss of function of FBXW7 or BTRCP lead to the stabilization of their cognate substrate proteins, Notch, MYC and CyclinE or Beta Catenin, respectively, all of which are well known oncogenes [6], [9], [11], [12], [13], [14], [15].

The COP9 signalosome is a mega-dalton sized complex consisting of at least eight proteins originally identified through a genetic screen in Arabidopsis [16], [17]. The COP9 complex is known to regulate a variety of ubiquitin ligase complexes. The exact mechanism by which COP9 regulates the function of ubiquitin ligase complexes is not completely understood, but has been suggested to regulate the interaction between f-box proteins and Skp1 by regulating the conjugation of the small protein nedd8 to Cullin1 [18], [19]. The cycle of neddylation/de-neddylation facilitates the ubiquitin ligase-substrate interaction and subsequent turn-over of the substrate. Thus, the activity of many of these ubiquitin ligase complexes has been shown to require the interaction with the COP9 signalosome for proper function. Interestingly, multiple components of the COP9 signalosome are known to have COP9-independent functions and have been shown to contribute to cancer [20], [21], [22].

The FBXW4 locus was originally mapped as the region on human chromosome 10 that was the causal locus in the limb malformation disorder split hand and foot 3 (SHFM3) [23], [24], [25], [26]. SHFM3 is a defect in the development of the apical ectodermal ridge during limb formation that causes aplasia of the central digits leading to, in the most severe cases, only two digits per limb [27], [28]. Subsequently, it was also found that Fbxw4 was also the locus responsible for a spontaneous mouse developmental defect that resembled SHFM3 in humans [29]. An array of publications claiming that alteration of FBXW4 is responsible for the defects, followed by publications that suggest the upstream locus encoding Fgf8 may be the culprit [30], [31]. To date, no satisfactory data has unclouded the issue.

Irrespective of whether FBXW4 causally contributes to SHFM3, to date no molecular or biochemical function has been ascribed to FBXW4. By combining data mining, expression studies, proteomics and biochemistry we have begun to expand our knowledge of FBXW4. We demonstrate that Fbxw4 is part of a ubiquitin ligase complex containing Skp1, Cullin1, Rbx1 and the COP9 signalosome. Assembly of this complex is dependent on the F-box domain of Fbxw4. Importantly, we show that FBXW4 locus is commonly deleted, under-expressed and somatically mutated in human cancers. Furthermore decreased FBXW4 expression correlates with poor survival of non-small cell lung cancer patients. Taken together, we hypothesize that FBXW4 may be an unappreciated tumor suppressor in human malignancies by virtue of its ability to regulate the function of critical signaling pathways.

## Materials and Methods

### rt-PCR analysis of Fbxw4 expression

qrt-PCR was performed on the ‘mouse normal tissue qPCR panel I’ from Origene cat. #MNRT101 (Rockville, MD, USA) using Sybr Green from Applied Biosystmes (Foster City, CA, USA) with the provided GAPDH oligos and ‘3 mus Fbxw4 rt’ 5′-GTCCTCATCATGCCCAGAGAAGAC-3′ with ‘5 mus Fbxw4 atg’ 5′-ATGGCGGAGGACGCGGCGGAGGATGC-3′ or ‘5 mus Fbxw4 rt’ 5′- GTCCTGTG GTTATGACACCTATG-3′ and ‘3 mus Fbxw4 no stop’ 5′-TGGGTTCTGA AAGTCTAAGACGTG-3′.

### Plasmid constructs

Fbxw4 and Fbxo46 constructs were purchased from Origene (Rockville, MD, USA) and used as PCR templates. (Fbxw4 cat. #MMM1013-7512491; Fbxo46 cat. #MMM98477851). Fbxo46 was amplified with the following oligos using Vent DNA polymerase (NEBL, Ipswich, MA, USA): mus Fbxo46 atg RI, 5′- GCGCGAATTCACCATGGACAGGGGCAGCCTCCTGCCC-3′; mus Fbxo46 no stop SalI, 5′- GCGCGTCGACCCTCCCCTCTTCCCGGCCAGC-3′. PCR amplified fragments were cloned into TOPO blunt cloning kit (Invitrogen, Carlsbad, CA, USA). The full-length Fbxo46 was then digested with EcoRI and SalI and cloned into pBABE-FLAG vector, digested EcoRI and XhoI, that contains a 3′ FLAG epitope tag down stream and in frame from the XhoI site, the FLAG epitope is then followed by stop codons and a SalI site. Fbxo46 with a 3′ in-frame FLAG tag was then digested out of pBABE with EcoRI and SalI and cloned into the EcoRI and XhoI site of the retroviral vector MIGRX.

Fbxw4 was amplified from the origene clone with Vent DNA polymerase with the following oligos: mus Fbxw4 atg RI, 5′- GCGCGAATTCACCATGGCGGAGGACGCGGCGGAGGATG-3′; mus Fbxw4 flag stop xhoI, 5′- GCGCCTCGAGTTACTTATCGTCGTCATCCTTATAATCCATTGGGTTCTGAAAGTCTAAG-3′. PCR amplified fragments were cloned into TOPO blunt. Full length Fbxw4 containing an in-frame 3′ FLAG tag was then recovered by digesting EcoRI and XhoI. This fragment was then cloned into the EcoRI and XhoI sites of MIGRX.

Fbxw4^−fbox^ was amplified from the full-length Fbxw4 TOPO clone using the following oligos: Fbxw4 flag stop xhoI, 5′- GCGCCTCGAGTTACTTATCGTCGTCATCCTTATAATCCATTGGGTTCTGAAAGTCTAAG-3′, mus Fbxw4 –fbox EcoRI, 5-GCGCGAATTCACCATGGCCCGGGCCTCGCTCAACACC-3′. The PCR amplified fragment was cloned into the EcoRI and XhoI sites of MIGRX.

pRK5-HA-Ubiquitin Addgene #17608 deposited by Ted Dawson [32].

### Cell culture

293 T cells were acquired from ATCC (Manassas, VA, USA) and cultured in DMEM supplemented with 10% FBS. DNA transfections in 293 T cells were done using PEI, at a ratio of 2.5 micrograms of PEI/microgram of DNA. All cell extracts were prepared following scrape harvesting of 293 T cells using CHAPS lysis buffer (1% CHAPS detergent, 150 mM NaCl, 50 mM Tris pH 7, 5 mM EDTA). Protein concentrations were determined using BCA protein assay reagent from Thermo Scientific (Rockford, IL, USA) cat# 23225. 30 µg of total protein was used for standard western blot procedure. Chemiluminescent detection was performed using SuperSignal WestFemto from Thermo Scientific (Rockford, IL, USA) according to manufacturer's protocol.

### Immunoprecipitations

200 ug of protein was incubated in 400 ul of total CHAPS buffer and incubated with 15 ul of indicated affinity matrix for one hour at 4 degrees Celsius. Following incubation, the matrix was washed three times in CHAPS buffer and then SDS loading buffer was added directly to washed matrix, boiled, and loaded directly into the wells of a PAGE gel. Drug treatments were performed as described in the text using 25 uM MG132.

### Antibodies

Tubulin #B512, FLAG M2 conjugated agarose beads, FLAG poly-clonal #F7425 (Sigma, St. Louis, MO, USA); HA affinity matrix and HA #3F10 (Roche, Indianapolis, IN); Ubiquitin #3933 (Cell Signaling Technologies, Beverly, MA, USA); SKP1 cat. #ab10546 and COPS2/TRIP15 cat. #ab4537 (Abcam, Cambridge, MA, USA); COPS5/JAB1 cat. #sc-9074 (Santa Cruz biotechnology, Santa Cruz, CA, USA).

### Immunoprecipitation and Protein Identification by nano-Liquid Chromatograpy couple to Tandem Mass Spectrometry (LC-MS/M)

10–150 mm plates of 293 t cells were transiently transfected using Fugene6, as per the manufacturer's protocol (Roche, Indianapolis, IN). 48 hours post transfection the cells were harvested by scraping in the cold. CHAPS lysates were prepared and quantitated as described above. 50 mg of total lysate were incubated with FLAG conjugated agarose beads (Sigma, St. Louis, MO, USA) over night at 4 degrees with gentle rocking. Agarose beads were added to a column and washed with 10 times the column volume by gravity flow. Column was capped and 1 bead bed volume of FLAG peptide (dissolved in PBS at a concentration of 5 mg/ml and then diluted 1∶10 in CHAPS buffer) was added to the column and incubated for 30 minutes at 4 degrees. The cap was removed and ∼100 ul fractions were collected as the elution flowed through by gravity. 50% of each fraction (∼5 fractions) was western blotted with anti-FLAG antibody. Typically the first fraction was devoid of the target protein and either fraction 2 or 3 had the bulk of target protein. ∼40% of the fraction containing the majority of FLAG-tag purified protein was partially resolved using SDS-polyacrylamide gel electrophoresis; the mixtures were concentrated into a single, 3-mm wide “stack” by electrophoresing through an SDS ‘stacking gel’ until entering the ‘separation gel’, followed by brief staining with Coomassie Blue and excision of the stacked protein band. Sample preparation and mass spectrometry was performed exactly as described previously [33].

Scaffold (Proteome Software Inc., Portland, OR), version 3_6_1 was used to further validate and cross-tabulate the MS/MS-based peptide and protein identifications. Protein and peptide probability was set at 95% with a minimum peptide requirement of one.

## Results

### Fbxw4 is a common site of proviral insertion and is expressed variably in normal murine tissues

**Figure 1:**
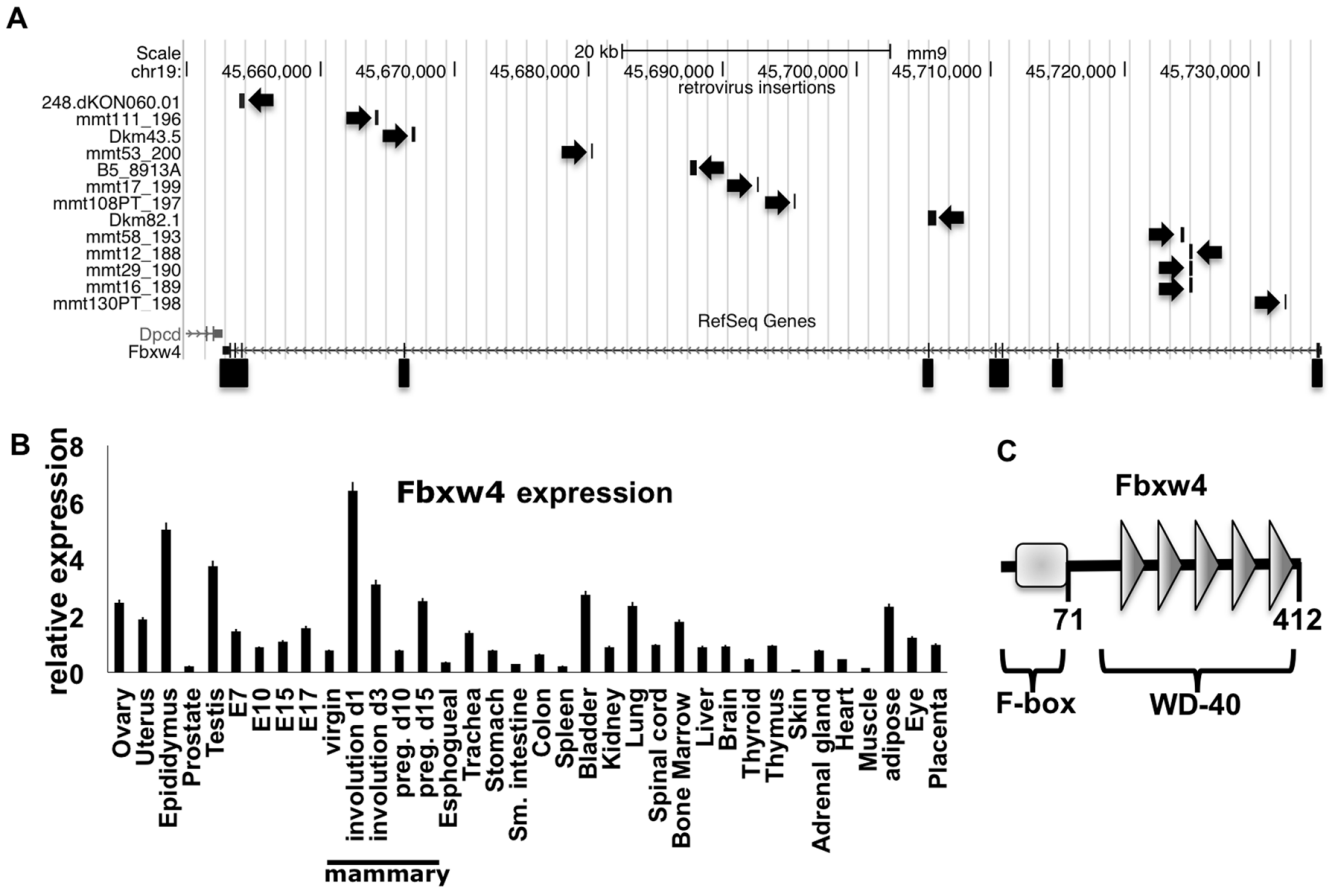
The Fbxw4 locus is a common site of proviral insertion and is highly expressed in the murine mammary gland during involution. A. Examination of the UCSC genome browser (genome.ucsc.edu) and the Retroviral Tagged Cancer Gene Database (http://variation.osu.edu/rtcgd/index.html) shows that multiple retroviral insertions have been cloned from within the transcribed Fbxw4 locus. Arrows indicate the directionality of the inserted provirus. The exons are indicated at the bottom and the position and scale of murine chromosome 19 is shown on top. Names given to the cloned proviral insertions are indicated on the left. Insertion sites beginning with ‘mmt’ were cloned from mouse mammary tumor virus induced mammary carcinomas. Insertion sites beginning with ‘Dkm’, ‘248’ and ‘B5’ were cloned from leukemias from murine leukemia virus accelerated hematopoietic cancers. B. FBXW4 is variably expressed in normal murine tissues. Oligos specific for murine Fbxw4 were used to perform quantitative rt-PCR on the ‘mouse normal cDNA TissueScan array’. Values were normalized to quantitative rt-PCR for GAPDH. C. Schematic of the Fbxw4 protein. Numbers represent the amino acids of the protein. Domains contained within Fbxw4 are indicated.

The identification and characterization of novel genes and gene products has led to an overwhelming amount of publicly available data and datasets. We wondered if mining these available datasets could produce hypothesis-generating observations surrounding potentially interesting genes. One such database is the ‘retroviral tagged cancer gene database’, which is a repository of proviral insertion sites clones from various investigators searching for novel genes that can contribute to leukemogenesis (and more recently transposon-mediated mutagenesis studies in hematopoietic and solid malignancies). Common sites of proviral integration are loci that provide a selective advantage to the cells by disregulating the expression of cellular genes. As support, most of the commonest sites of proviral insertion are bona fide oncogenes or tumor suppressors. We queried this database and found that the Fbxw4 gene locus was the only locus in the 30 most commonly identified insertion loci that has not been suggested to play a role in cancer or been biochemically characterized. There have been, to date, 13 insertion sites cloned from within the encoded Fbxw4 gene and insertions have been cloned in both forward and reverse orientation (Figure 1A). This data may suggest that proviral insertion into the locus can lead to loss of Fbxw4 function.

The fact that Fbxw4 has never been characterized prompted us to examine the expression of this locus in more detail. First we determined what the expression pattern of Fbxw4 mRNA is across normal murine tissues. Using cDNA prepared from various mouse tissues we observed a striking level of expression specifically in the involuting mammary gland (Figure 1B). This suggests the possibility that Fbxw4 expression is increased and may contribute during an apoptotic process.

### Fbxw4 interacts with components of an E3 ubiquitin ligase and the COP9 signalosome

**Figure 2:**
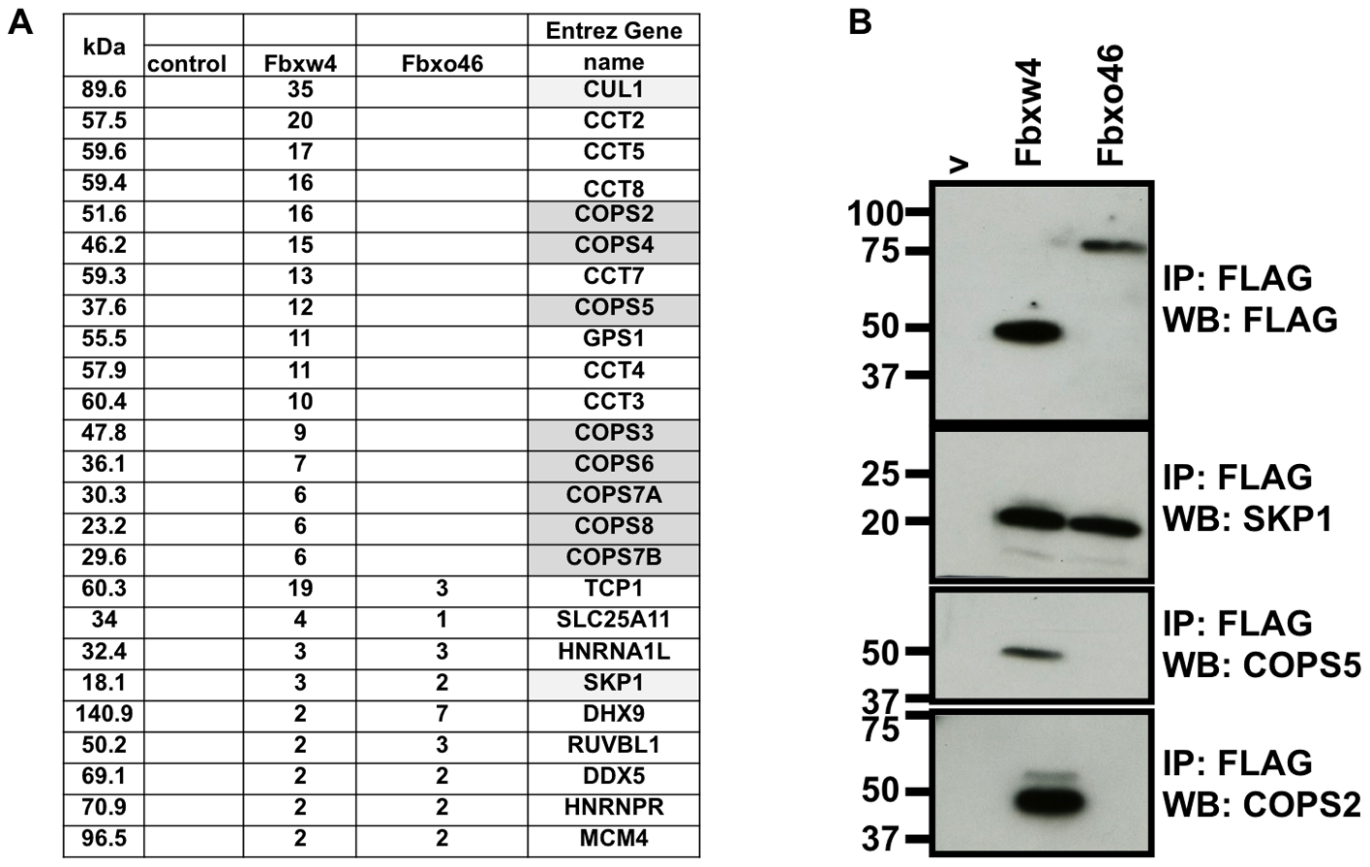
Fbxw4 interacts with components of an E3 ubiquitin ligase complex and the COP9 signalosome. A. Table representing the number of unique peptides identified from one representative mass spectrometry experiment following FLAG immunoprecipitation from lysates of control cells expressing FLAG only “contr.”, or cells expressing FLAG-Fbxw4 or FLAG-Fbxo46. Column on left indicates the size of the interacting protein, in kilo-daltons (kDa). Gene names of proteins that contain the identified peptides are shown in the right column. Components of an E3 ubiquitin ligase complex are shaded in light gray; components of the COP9 signalosome are shaded dark gray. B. Validation of data mass spectrometry data by immunoprecipitation followed by western blot. 293 T cells were transfected with plasmids containing FLAG- Fbxw4, FLAG-Fbxo46 or an empty vector (v). 48 hours post-transfection cell lysates were prepared and immunoprecipitations were performed with mono-clonal anti-FLAG antibodies (M2) (to immunoprecipitate Fbxw4- or Fbxo46-interacting complexes). Western blots were performed to detect Fbxw4 or Fbxo46 (FLAG rb; polyclonal FLAG antibody; top panel), SKP1, COPS5, or COPS2. C. Expression of Fbxw4 alters the migration of endogenous SKP1 by gel filtration chromatography. 293 T cells were transfected with an empty vector (left panels) or a cplasmid containing FLAG-Fbxw4 (right panels). 48 hours post-transfection cell lysates were prepared and separated on a superpose6 gel filtration column. Western blots were performed on every other fraction to detect Fbxw4 (top panels) or SKP1 (bottom panels). In the absence of Fbxw4 SKP1 elutes with a peak at fraction 23, whereas when Fbxw4 is expressed there is co-elution of Fbxw4 with peaks at fraction 15 and in the void volume. Size standards that elute from given fractions are shown.

A protein motif algorithm shows that the Fbxw4 protein contains an F-box domain, a motif that is usually implicated in interactions with an E3 ubiquitin ligase complex and five WD-40 motifs, which are know protein-protein interaction modules (Figure 1C). Despite the genetic work performed on the Fbxw4 locus, there have been no studies examining the biochemical function of Fbxw4. As a first attempt to understand the possible function of Fbxw4 we performed immunoprecipitation on FLAG-tagged Fbxw4 followed by mass spectrometry to identify, in an unbiased manner, Fbxw4 interacting proteins (Figure 2A). Two controls were performed to aid in interpretation of the data; immunoprecipitation of cell lysates expressing only the FLAG epitope tag (cont.) or immunoprecipitation of cell lysates expressing a FLAG-tagged F-box “only” containing protein (Fbxo46) were performed. Data from these experiments showed that Fbxw4, and not control, immunoprecipitated components of an E3 ubiquitin ligase (SKP1 and CUL1) and the components of the COP9 signalosome, whereas both F-box containing proteins interacted with SKP1. Interestingly, one peptide corresponding to RBX1 was also identified in the FLAG-immunoprecipitation from Fbxw4 expressing lysates and not from the other lysates (not shown)

To validate the data from the mass spectrometry we performed immunoprecipitations followed by western blots with antibodies specific to the identified proteins. New lysates were prepared that expressed either FLAG-Fbxw4, FLAG-Fbxo46, or FLAG only control (Figure 2B). Data obtained demonstrate that both Fbxw4 and Fbxo46 interact with SKP1, but only Fbxw4 interacts with components of the COP9 signalosome.

To further strengthen the finding that Fbxw4 can interact with endogenous SKP1 we performed gel filtration chromatography on cell lysates transfected with an empty vecotor or a vector expression FLAG-Fbxw4 (Figure 2C). Fractions obtained from the chromatography were western blotted with anti-SKP1 antibody. Cells that do not express exogenous Fbxw4 contain SKP1 that elutes in a fraction corresponding to less than 67 kDa, whereas expression of Fbxw4 leads to co-elution of SKP1 with Fbxw4 in two new complexes of over 200 kDa and a complex that elutes in the void. The altered migration of SKP1 into two new fractions that co-elute with Fbxw4 supports the finding the Fbxw4 interacts with components of an E3 ligase complex.

### Fbxw4 interacts with SKP1 and the COP9 signalosome in an F-box dependent manner

**Figure 3:**
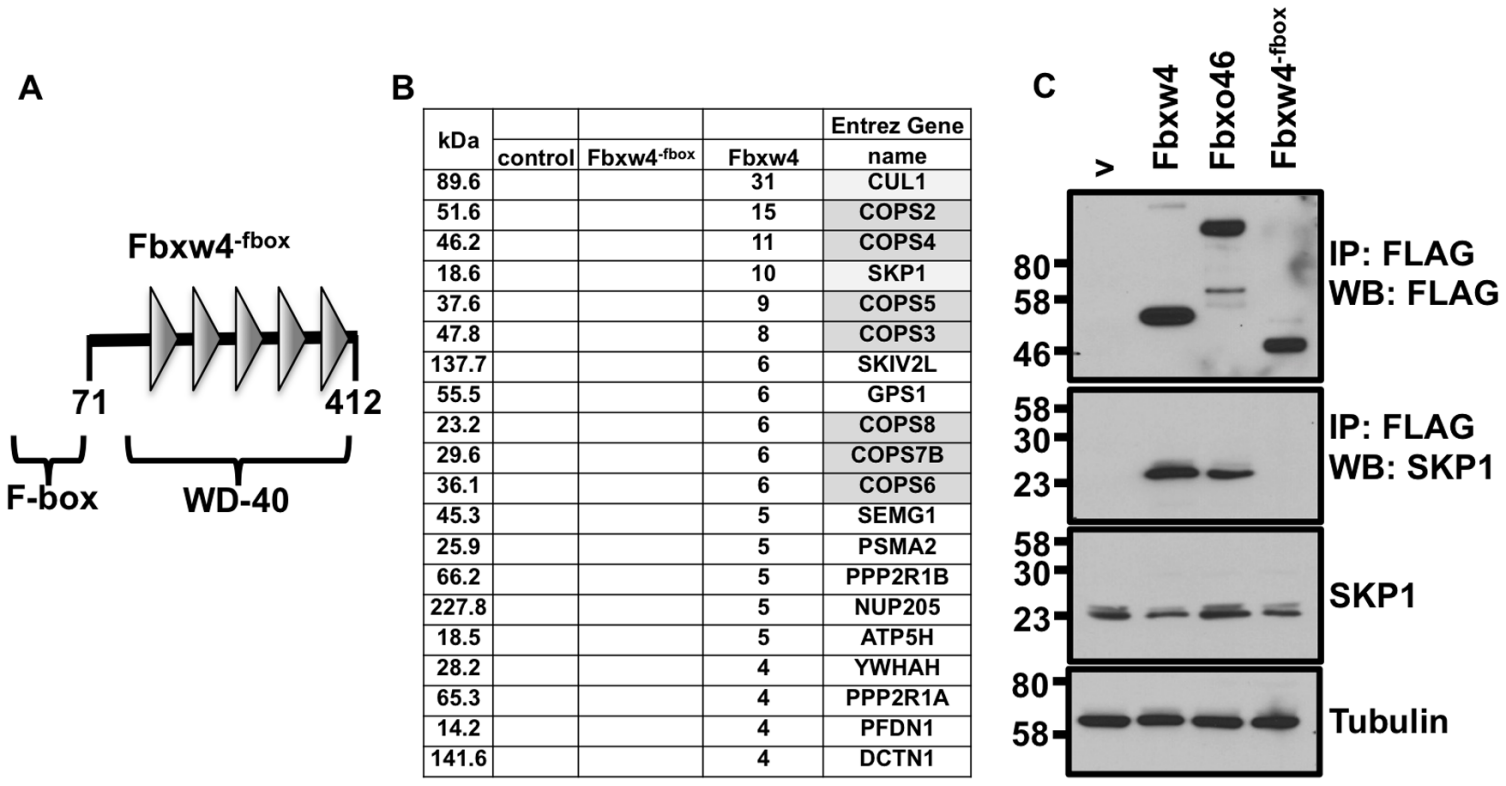
Fbxw4 interacts with E3 ubiquitin ligase components and with the COP9 signalosome in an F-box dependent manner. A. Schematic of the Fbxw4^−fbox^ protein. Numbers represent the amino acids of the protein. Domains contained within Fbxw4^−fbox^ are indicated. B. Table representing the number of unique peptides identified from one representative mass spectrometry experiment following FLAG immunoprecipitation from lysates of control cells expressing FLAG only “contr.”, or cells expressing FLAG- Fbxw4 or FLAG- Fbxw4^−fbox^. Column on left indicates the size of the interacting protein, in kilo-daltons (kDa). Gene names of proteins that contain the identified peptides are shown in the right column. Components of an E3 ubiquitin ligase complex are shaded in light gray; components of the COP9 signalosome are shaded dark gray. C. Validation of data mass spectrometry data by immunoprecipitation followed by western blot. 293 T cells were transfected with plasmids containing FLAG-Fbxw4, FLAG-Fbxw4^−fbox^, FLAG-Fbxo46 or an empty vector (v). 48 hours post-transfection cell lysates were prepared and immunoprecipitations were performed with mono-clonal anti-FLAG antibodies (M2) (to immunoprecipitate Fbxw4-, Fbxw4^−fbox^-, or Fbxo46-interacting complexes). Western blots were performed to detect Fbxw4, Fbxw4^−fbox^ or Fbxo46 (FLAG rb; polyclonal FLAG antibody; top panel) or SKP1.

To begin to determine the precise biochemical function of Fbxw4, we wanted to know which interactions depended on the F-box domain. To this end, we engineered an FBXW4 construct that lacks the first 71 amino acids, termed Fbxw4^−fbox^ (Figure 3A). Again, immunoprecipitation followed by mass spectrometry was performed from cell lysates expressing FLAG-Fbxw4, FLAG- Fbxw4^−fbox^, or FLAG only, to determine what proteins interact with the respective proteins. No peptides corresponding to components of the E3 ubiquitin ligase or components of the COP9 signalosome were identified with Fbxw4^−fbox^, whereas these proteins were once again found to interact with Fbxw4 (Figure 3B). To validate the data from the mass spectrometry we performed immunoprecipitations followed by western blots with antibodies specific to the identified Fbxw4 interacting proteins. Lysates were prepared that expressed either FLAG-FBXW4, FLAG- Fbxw4^−fbox^, FLAG-Fbxo46, or FLAG only control. Data show that Fbxw4 and Fbxo46, but not Fbxw4^−fbox^ interact with SKP1 (Figure 3C).

### FBXW4 interacts with ubiquitinated cellular proteins

**Figure 4:**
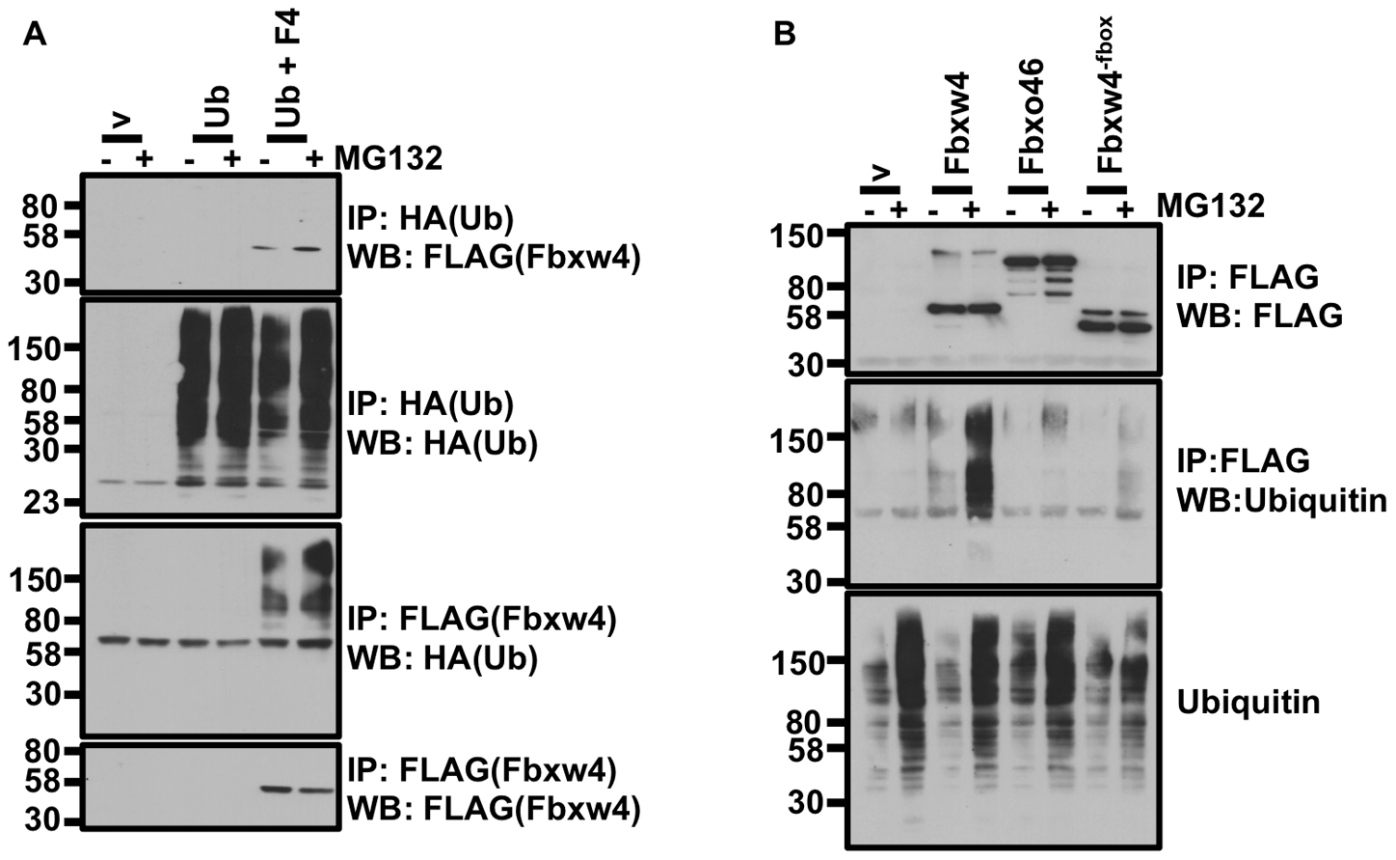
FBXW4 associates with ubiquitinated cellular proteins. A. Fbxw4 can be immunoprecipitated by ubiquitinated proteins and Fbxw4 can immunoprecipitate ubiquitinated proteins. 293 T cells were transfected with empty vector (v), HA-Ubiquitin, or HA-Ubiquitin and FLAG-Fbxw4. 36 hours post-transfection cells were treated with MG132 (+) or left untreated (−) for six hours. Cell lysates were prepared and immunoprecipitations were performed with either anti-HA antibodies (top two panels) or mono-clonal anti-FLAG antibodies (M2) (bottom two panels). Western blots were performed on both sets of immunoprecipitations with anti-FLAG and anti-HA antibodies. B. FBXW4 associates with cellular proteins that are endogenously ubiquitinated. 293 T cells were transfected with plasmids containing FLAG-Fbxw4, FLAG- Fbxw4^−fbox^, FLAG-Fbxo46 or an empty vector (v). 36 hours post-transfection cells were treated with MG132 (+) or left untreated (−) for six hours. Cell lysates were prepared and immunoprecipitations were performed with mono-clonal anti-FLAG antibodies (M2) (to immunoprecipitate Fbxw4-, Fbxw4^−fbox^-, or FBXO46-interacting complexes). Western blots were performed to detect Fbxw4, Fbxw4^−fbox^ or Fbxo46 (FLAG rb; polyclonal FLAG antibody; top panel) or Ubiquitin.

We have conclusively demonstrated that Fbxw4 interacts with an E3 ubiquitin ligase complex and the COP9 signalosome. We posited that Fbxw4 would interact with ubqiuitinated proteins within the cell. It is known that for many substrates of E3 ubiquitin ligases, ubiquitination of the substrate protein leads to rapid release from the complex. We therefore hypothesized that inhibition of the proteasome would lead to an accumulation of those cellular proteins ubiquitinated by the Fbxw4 ligase complex. Thus, accumulation of ubiquitinated proteins would, in turn, lead to enrichment of the interactions between Fbxw4 and ubiquitinated substrates. Cells were transfected with an empty vector (v), HA-Ubiquitin, or HA-Ubiquitin and FLAG-Fbxw4. 36 hours post-transfection cells were treated with the proteasome inhibitor, MG132, for six hours. Cell extracts were prepared and immunoprecipitated with anti-HA antibody followed by western blot for Fbxw4 (Figure 4A, top panel). An increased amount of Fbxw4 was found to interact with ubiquitinated proteins following MG132 treatment. This Fbxw4 appeared to be the normal molecular weight suggesting an increased association of FBXW4 with ubiquitinated proteins and not an increase in ubiquitated Fbxw4. Furthermore, the increased amount of Fbxw4 that immunoprecipitated with anti-HA antiobody was not due to an increase in the total amount of Fbxw4 since there was a slight decrease in the total amount of Fbxw4 following MG132 treatment (Figure 4A, lower panel). As further support of this notion, there was an increase in the total levels of ubiquitinated proteins and an increase in the amount of ubiquitinated proteins that immunoprecipitated with Fbxw4 following immunoprecipitation of Fbxw4 from the same lysates following MG132 treatment (Figure 4A, middle panels).

We wanted to know whether similar results would be obtained looking at endogenous ubiquitin, as opposed to over-expressed HA-Ubiquitin. In addition, we wanted to determine whether the previously observed results were dependent on an intact F-box domain. To this end, cells were transfected with FLAG-Fbxw4, FLAG- Fbxw4^−fbox^, FLAG-Fbxo46, or FLAG only control vector (v). Cells were either treated with vehicle or MG132 for six hours and cell lysates were prepared. Immunoprecipitation with anti-FLAG antibodies were performed and western blots were performed to detect endogenous ubiquitin or FLAG-tagged proteins. We observed a dramatic increase in the amount of ubiquitin that associated with Fbxw4. This result was not simply due to over-expression of Fbxw4 since neither Fbxw4^−fbox^ or Fbxo46, which were expressed to higher levels, demonstrated an increased interaction with ubiquitinated proteins following MG132-induced inhibition of the proteasome. These data indicate that an intact F-box domain is required for association of Fbxw4 with ubiquitinated proteins and not all F-box containing proteins are capable of such dramatic increased interactions with ubiquitinated cellular proteins following inhibition of the proteasome. Although the interaction between Fbxw4 and cellular ubiquitinated proteins is dependent on an in tact f-box domain more work is required to determine if these proteins are bona fide substrates of the SCFFbxw4 ubiquitin ligase complex or simply proteins that interact with the complex through alternative mechanisms.

### FBXW4 is mutated, lost and under-expressed in human cancers

**Figure 5:**
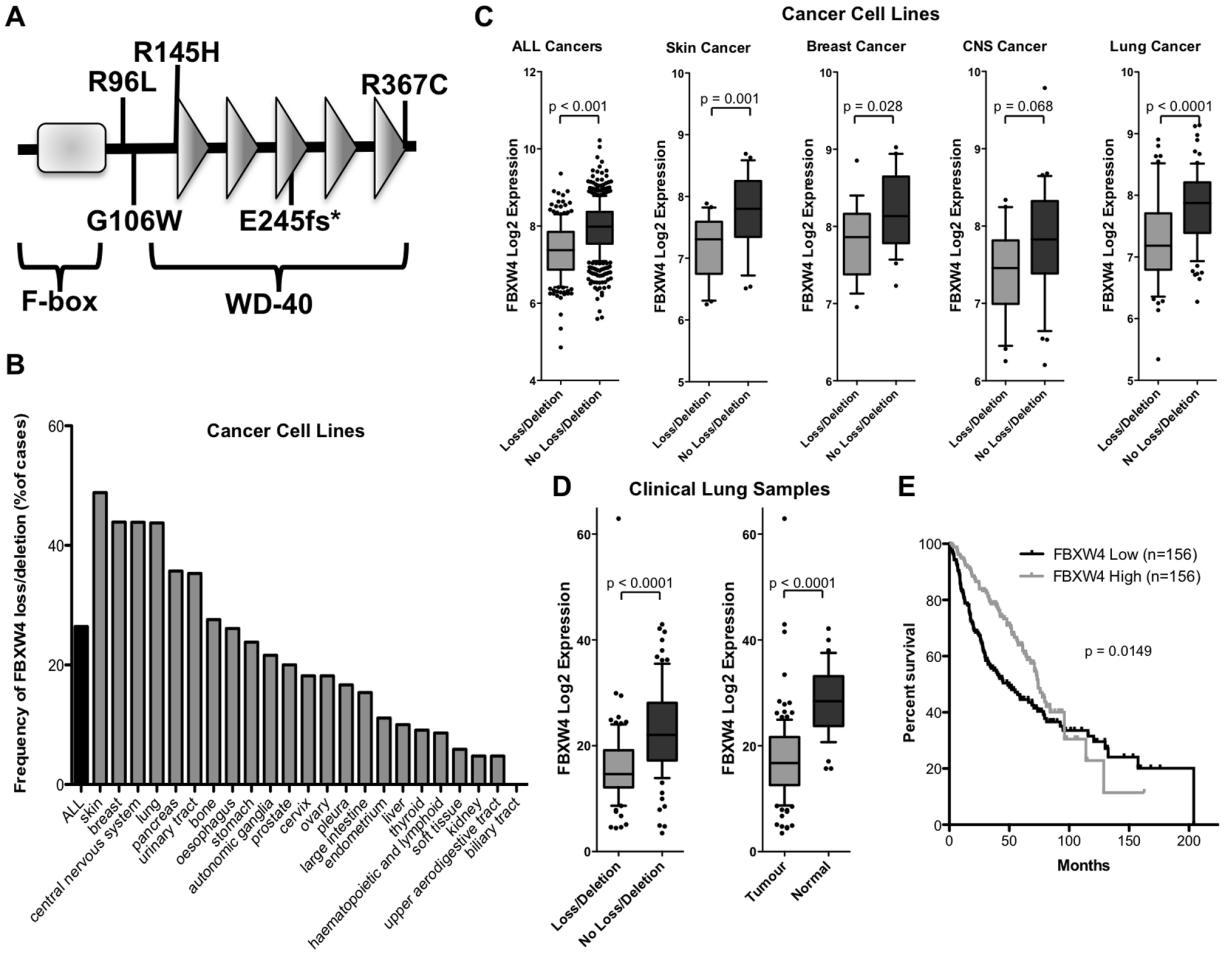
FBXW4 is mutated, lost and under-expressed in human cancers and associated with poor survival in lung adenocarcinoma. A. FBXW4 is somatically mutated in human cancers. The Cancer Genome Atlas (TCGA) project and the Catalogue of Somatic Mutations in Cancer (COSMIC) from the Sanger Institute were queried for somatic mutations in FBXW4. Of ∼470 tumors that were analyzed for mutation in FBXW4, five somatic mutations were observed at the amino acids indicated on the schematic. Interestingly, the four single amino acids that are targets of mutations (R96, G106, R145 and R367) are evolutionarily conserved in Drosophila. R96L, G106W and R367C are predicted to disrupt protein function (significance p = <.0003, using ProPhylER algorithm) and the E245fs* mutation produces a protein that lacks the last two and a half WD-49 motifs. B. FBXW4 is frequently lost in human cancers. The frequency of DNA copy number loss/deletion across 719 human cancer cell lines representing diverse tissues of origin from the Sanger Institutes Cancer Genome Project are presented along with the frequency in the individual cancer types with n ≥ 5. C. FBXW4 is underexpressed in cancer cell lines with copy number loss. Box plots illustrating the mRNA expression levels for cell lines with loss/deletion and those without loss/deletion for all cancer cell lines from B. with corresponding gene expression microarray profiles are presented along with expression in the four individual cancer types with the highest frequency of loss/deletion (skin, breast, central nervous system (CNS) and lung cancer). Expression levels were compared between the two groups using the Mann-Whitney U-test and a p<0.01 was considered significant. Whiskers represent the 10–90 percentiles with dots displaying the outliers. D. FBXW4 is lost and under-expressed in clinical lung adenocarcinoma tumors from TCGA project. The expression of FBXW4 (RNA-seq RPKM values) is plotted for tumors with loss/deletion vs those without loss/deletion (left panel) as well as tumors and normal lung cancer tissue (right panel). Expression levels were compared between the groups and presented as in C. with a p<0.01 considered significant. E. Low FBXW4 expression is associated with poor survival in lung adenocarcinoma patients. Lung adenocarcinoma patients from the Directors Challenge dataset were stratified into tertiles based on their FBXW4 expression levels and the survival times between the bottom and top tertiles were compared using the Log-rank (Mantel-Cox) Test.

To begin to address the possibility that FBXW4 is important in human cancer we examined whether the gene is mutated in human cancers. By querying available public databases (The Cancer Genome Atlas (TCGA) project and the Catalogue of Somatic Mutations in Cancer (COSMIC) from the Sanger Institute) we found that FBXW4 is somatically mutated in seven of ∼470 tumors that have been sequence, thus far (Figure 5A). Two of the mutations are silent and do not lead to changes in the amino acid sequence of the protein, but interestingly, the four missense mutations (R96, G106, R145 and R367) are evolutionarily conserved all the way down to some species of Drosophila. In fact, R96L, G106W and R367C were predicted to disrupt protein function with a very high significance (p = <.0003) using ProPhylER algorithm. In addition to these predicted detrimental mutations, a frame shift mutation, E245fs*, was observed in a breast carcinoma patient that would lead to the production of a FBXW4 protein that is lacking the last three WD-40 motifs (Figure 5A). The finding that somatic mutations found in human cancers are predicted to disrupt critical aspects of FBXW4 function strengthens the possibility that FBXW4 regulates important homeostatic processes.

Thus far, our data indicate that disruption of FBXW4 may be important in both murine proviral insertional mutagenesis studies and in human cancer patients. To extend these observations we sought to determine the frequency which FBXW4 locus is lost in human cancers. Again we turned to publicly available datasets and examined the frequency of FBXW4 DNA copy number loss/deletion across 719 diverse human cancer cell lines (Sanger Institutes Cancer Genome Project). The FBXW4 locus is lost or deleted in over 25% of all the cell lines examined with some cancer cell types displaying loss in more than 40% of the lines (skin, breast, central nervous system and lung) (Figure 5B). Importantly, if a particular locus is lost in cancer cells, there may be mechanisms by which the remaining allele can compensate, such that the total level of mRNA produced is not decreased although one of the two alleles is lost. Therefore, we also examined the expression levels of FBXW4 mRNA in cancer cell lines that have lost at least one copy of the FBXW4 gene, compared to cell lines where both copies are seemingly intact. We found that FBXW4 mRNA expression is dramatically and significantly decreased across all lines that have lost the FBXW4 locus (Figure 5C). This was also found to be true when the cancer types with the highest frequency of FBXW4 loss were separated and examined separately. To ensure that the above observations were not due to the analysis of cultured human cell lines, we also examined clinical lung adenocarcinoma tumors from TCGA project. The expression of FBXW4, as determined by next generation RNA-sequencing was compared between tumors with loss/deletion of FBXW4 with those without loss/deletion. As before, there was a significant decrease in FBXW4 mRNA in tumors that have lost at least one allele of the locus (Figure 5D). Furthermore, when the expression of FBXW4 mRNA in all lung cancers was compared with the expression in normal lung controls there was a significantly lower level of FBXW4 in cancer samples. Finally, we wanted to determine if the expression of FBXW4 mRNA could be a prognostic indicator for the survival outcome of human patients with lung cancer. Lung adenocarcinoma patients from the Directors Challenge dataset were stratified into tertiles based on their FBXW4 expression levels. The highest and lowest FBXW4 mRNA expressing tertiles were compared for survival outcome (Figure 5E). We observed a significant survival disadvantage for the patients expressing lower levels of FBXW4. The compilation of these data suggest that FBXW4 may act to keep cells from becoming malignant or may act to keep malignant cells from becoming more aggressive in patients.

## Discussion

The possibility of using publicly available datasets for the identification of novel, biologically important proteins has become readily possible. However, one of the major set-backs to such database mining is the inability to assign biochemical and biological functions to identified proteins. Herein, we have identified and characterized a previously unstudied protein, Fbxw4. By combing available databases, such as the retroviral cancer gene database, somatic mutation repositories, and expression data sets, we have found that the Fbxw4 locus in mouse is a common target of proviral insertional mutagenesis and FBXW4 is somatically mutated, lost and underexpressed in human cancers. Futhermore, we have used a number of biochemical approaches to determine that Fbxw4 interacts with SKP1, CUL1 and the COP9 signalosome in an f-box dependent manner. The studies presented demonstrate that Fbxw4 is an interesting and novel protein that may play a role in tumorigenesis.

The finding that Fbxw4 interacts with a ubiquitin ligase complex and that disruption of this complex may play a role in cancer progression is not an unfounded model. In fact, a number of ubiquitin ligase complexes are known to be bona fide tumor suppressors [6], [9], [12], [15]. In many cases, loss or mutation of an F-box containing protein leads to the aberrant expression of an oncogene. Such is the case for FBXW7, whereby mutation or loss of FBXW7 is found in high percentage of T-cell acute lymphoblastic leukemias [34]. An FBXW7-containing E3 ubiquitin ligase complex is known to target oncogenes such as Notch, MYC and CyclinE [6], [9], [12], [15]. Through our proteomics based approaches we have begun to attempt to identify the substrates proteins that are recognized and subsequently targeted for degredation by Fbxw4-containing ubiquitin ligase complexes. We hypothesize that Fbxw4 interacts with proteins known to modulate aspects of cell proliferation or cell survival, however more work is required.

Previous work on FBXW4 centered around a controversy that originally began with the identification of a limb development defect, split hand and foot malformation (SHFM) in mouse and man [23], [24], [25], [26]. The gene locus, originally called Dactylin, that was associated with a defect in the development of apical ectodermal ridge (AER) was mapped to human chromosome 10q24 where FBXW4 resides [25], [29]. Many publications suggested that alterations in the FBXW4 gene and/or its expression were the causal event leading to severe malformations in limb development [35], [36]. However, confounding the field was the identification of spontaneous mouse mutants, which had limb defects similar to those found in humans that harbored endogenous retro-transposons in the Fbxw4 gene [36]. In addition, it was found that proviral insertions in Fbxw4 in zebrafish caused a strip patterning defect [37]. The controversy has since evolved due to reports that Fbxw4 expression is not altered in these mice or in humans with SHFM, but rather expression of an upstream gene, Fgf8, may be altered [31]. Thus far, solid evidence has not been provided either genetically or biochemically, to sufficiently support or refute any of these claims. Herein, by characterizing the biochemical properties of Fbxw4 for the first time we hope to gain an understanding of the cellular signaling pathways regulated by Fbxw4 with the goal of shedding light on a role for Fbxw4 in normal development and in disease. To this end, future work will specifically examine interacting proteins that may be suspected to affect normal developmental programs. Furthermore, more in depth genetic analysis of Fbxw4 in murine development will extend our understanding for how this novel gene can regulate important signaling pathways.
